# Influence of the Bone Marrow Microenvironment on Hematopoietic Stem Cell Behavior Post-Allogeneic Transplantation: Development of Clonal Hematopoiesis and Telomere Dynamics

**DOI:** 10.3390/ijms251910258

**Published:** 2024-09-24

**Authors:** Myungshin Kim, Dain Kang, Hoon Seok Kim, Jong-Mi Lee, Silvia Park, Daehun Kwag, Chaeyeon Lee, Yuna Hong, Duyeon Na, Youngil Koh, Choong Hyun Sun, Hongyul An, Yoo-Jin Kim, Yonggoo Kim

**Affiliations:** 1 Catholic Genetic Laboratory Center, Seoul St. Mary’s Hospital, College of Medicine, The Catholic University of Korea, Seoul 06591, Republic of Korea; microkim@catholic.ac.kr (M.K.); oskitty@catholic.ac.kr (D.K.); hskim11@catholic.ac.kr (H.S.K.); jongmi1226@catholic.ac.kr (J.-M.L.); lcywin0813@catholic.ac.kr (C.L.); yuna1013@catholic.ac.kr (Y.H.); msna09@catholic.ac.kr (D.N.); 2 Department of Laboratory Medicine, College of Medicine, The Catholic University of Korea, Seoul 06591, Republic of Korea; 3 Department of Hematology, Seoul St. Mary’s Hematology Hospital, College of Medicine, The Catholic University of Korea, Seoul 06591, Republic of Korea; silvia.park@catholic.ac.kr (S.P.); kdh@catholic.ac.kr (D.K.); 4 Leukemia Research Institute, College of Medicine, The Catholic University of Korea, Seoul 06591, Republic of Korea; 5 Department of Medical Sciences, Graduate School of The Catholic University of Korea, Seoul 06591, Republic of Korea; 6 Department of Internal Medicine, Seoul National University Hospital, Seoul 03080, Republic of Korea; go01@snu.ac.kr; 7 NOBO Medicine, Inc., Seoul 04799, Republic of Korea; denilsun@nobomedicine.com (C.H.S.); anhongyul@nobomedicine.com (H.A.)

**Keywords:** clonal hematopoiesis, telomere length, hematopoietic stem cell transplantation, bone marrow microenvironment, myelodysplastic neoplasms, somatic mutations, genetic evolution

## Abstract

Allogeneic hematopoietic stem cell transplantation (allo-HSCT) is a potential cure for myelodysplastic neoplasms (MDSs) and other hematologic malignancies. This study investigates post-transplantation genetic evolution and telomere dynamics in hematopoietic cells, with a focus on clonal hematopoiesis (CH). We conducted a longitudinal analysis of 21 MDS patients who underwent allo-HSCT between September 2009 and February 2015. Genetic profiles of hematopoietic cells from both recipients and donors were compared at equivalent pre- and post-transplantation time points. Targeted sequencing identified CH-associated mutations, and real-time quantitative PCR measured telomere length. Furthermore, we compared CH incidence between recipients and age-matched controls from the GENIE cohort from routine health checkups. Post-allo-HSCT, 38% of recipients developed somatic mutations not detected before transplantation, indicating de novo CH originating from donor cells. Compared to age-matched healthy controls, recipients showed a significantly higher incidence of CH, suggesting increased susceptibility to genetic changes post-transplant. Telomere length analysis also revealed accelerated shortening in transplanted cells, highlighting the heightened stress and proliferation demands in the new microenvironment. Our findings reveal a notable incidence of donor-derived CH in allo-HSCT recipients, alongside significant telomere attrition. This suggests the potential influence of the marrow microenvironment on genetic and molecular changes in hematopoietic cells.

## 1. Introduction

Myeloid neoplasms, such as myelodysplastic neoplasm (MDS) and acute myeloid leukemia, stem from the clonal malignant transformation of hematopoietic stem cells (HSCs), primarily driven by genetic mutations. Moreover, mounting evidence suggests a strong association between these diseases and the bone marrow (BM) microenvironment, particularly with mesenchymal stromal cells (MSCs) and their interaction with HSCs [1]. Research increasingly points to a significant role of MSCs in MDS development, implying that they, acting as a microenvironment, may not only be affected by the disease but could potentially initiate it [2,3,4]. Studies in mouse models have shown that deficiencies in osteoblasts derived from marrow stromal cells can induce MDS and secondary acute myeloid leukemia [5], highlighting the potential causal implications of the BM microenvironment in myeloid neoplasm pathology [6,7].

Allogeneic hematopoietic stem cell transplantation (allo-HSCT) is a crucial and potentially curative therapeutic option for hematological malignancies, including MDS. In allo-HSCT, healthy HSCs are obtained from donors and transplanted into recipients with the aim of achieving engraftment. During this process, the transplanted HSCs survive and proliferate, striving to establish hematologic homeostasis [8]. However, transplant procedures do not replace the recipient’s BM MSCs; instead, they may disrupt the BM microenvironment to some extent. Consequently, transplanted healthy HSCs might encounter a diseased or dysfunctional BM microenvironment, including the recipient’s MSCs. It is conceivable that genetically mutated clones within an HSC population might emerge and be selected due to this interaction with the diseased BM microenvironment [9]. In this context, allo-HSCT could provide a valuable model for analyzing how the BM microenvironment influences HSCs.

In this study, we aimed to assess the impact of the BM microenvironment on healthy HSCs by comparing genetic profiles of hematopoietic cells obtained from both recipients and their respective donors at equivalent time points before and after transplantation. This direct comparison enabled us to determine whether allo-HSCT in MDS patients generated and sustained vulnerable BM microenvironments conducive to genetic alterations and/or clonal selection

## 2. Results

### 2.1. Clinical Characteristics of Recipients

Disease and transplantation characteristics are presented in Table 1. The median age at HSCT for recipients was greater than for donors (44 vs. 39 years, *p* = 0.014). Additionally, the median age at study inclusion was greater for recipients compared to donors (50 vs. 45 years, *p* = 0.004). Fourteen recipients received HSCT from matched sibling donors, six from related haploidentical donors, and one from an unrelated donor. The median post-allo-HSCT follow-up duration was 6.0 years (range 2.5–8.4).

### 2.2. Mutations Identified before HSCT

**Figure 1:**
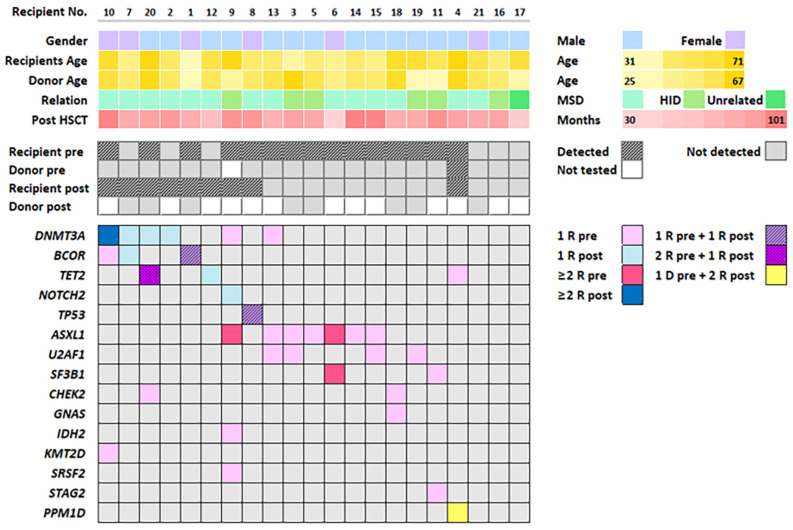
General characteristics. Abbreviations: No., number; HSCT, hematopoietic stem cell transplantation; pre, pre-HSCT; post, post-HSCT; MSD, matched sibling donor; HID, haploidentical donor; 1R, one mutation detected in recipient; 1D, one mutation detected in donor; 2D, two mutations detected in recipient; 2R, two mutations detected in donor; ≥2R, more than two mutations in recipient.

**Figure 2:**
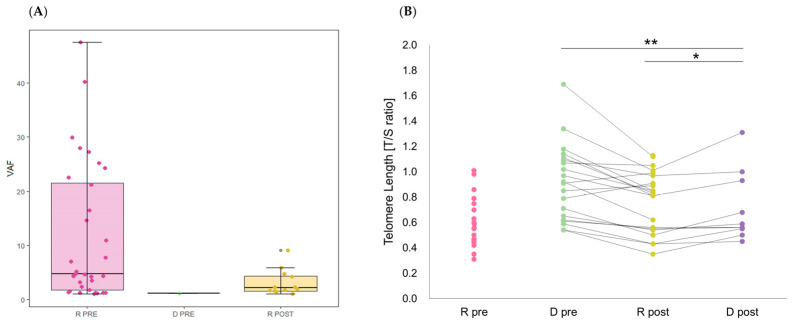
(**A**) The variant allele fractions (VAFs) of somatic mutations in recipients (R) and donors (D) before (pre) and after (post) hematopoietic stem cell transplantation. (**B**) Telomere length in individuals. * *p* < 0.05 and ** *p* < 0.01 by Wilcoxon signed-rank test.

Fifteen out of twenty-one recipients (71%) exhibited somatic mutations in their peripheral blood (PB) samples before HSCT. A total of 31 mutations were identified, with the number of mutations varying in each patient (median 2, range 1–5). The most frequently mutated gene was *ASXL1* (*n* = 9), followed by *U2AF1* (*n* = 4), *SF3B1* (*n* = 3), *TET2* (*n* = 3), *BCOR* (*n* = 2), *CHEK2* (*n* = 2), and *DNMT3A* (*n* = 2) (Figure 1). The variant allele fraction (VAF) varied, with a median of 4.78% (range 1.03–47.46%). The presence of mutations was not affected by bridging chemotherapy. Furthermore, among the twenty-one donors, only one exhibited a mutation in the PB sample before HSCT, involving the *PPM1D* gene with a low VAF (1.18%) (Figure 2).

### 2.3. Mutations Identified after HSCT

In post-allo-HSCT PB samples, eight recipients (38%) exhibited somatic mutations, with a median of 1 mutation per patient (range 1–3), resulting in a total of 12 identified mutations. The most frequently mutated gene was *DNMT3A* (*n* = 5), followed by *BCOR* (*n* = 2) and *TET2* (*n* = 2) (Figure 1). The median VAF was 2.00% (range 1.00–9.07%) (Figure 2). Notably, none of the mutations identified in post-allo-HSCT PB were consistent with those detected before HSCT. Intriguingly, the *PPM1D* gene mutation present in the donor’s PB persisted in the recipient’s body for 6 years with a slight increase in VAF to 1.52%. Out of the eight donors available for mutation analysis concurrent with the recipients’ post-allo-HSCT analyses, none exhibited mutations. Notably, three of the corresponding recipients showed mutations in post-allo-HSCT PB samples: *DNMT3A* and *BCOR* in one recipient, *DNMT3A* in another, and *TET2* in the third. This suggests that hematopoietic cells originating from the same individual but surviving in different bodies (donor vs. recipient) undergo distinct genetic evolution. Further details of detected mutations are summarized in Table S1.

We observed no differences in the presence of mutations after HSCT according to age at diagnosis, age at HSCT, or age at study inclusion. Similarly, there were no differences in the donor’s age at the time of HSCT and study inclusion. The use of ganciclovir to manage cytomegalovirus did not influence the presence of mutations. Furthermore, no significant differences were observed in gender, diagnosis, hypomethylating agent use, conditioning intensity, or the occurrence of acute or chronic GVHD based on the presence of mutations both before and after HSCT.

### 2.4. Higher Incidence of Clonal Hematopoiesis (CH) Development in Transplanted Hematopoietic Cells

After HSCT, with all recipients maintaining complete donor chimerism and a stable disease-free status, we considered mutations in post-allo-HSCT PB as CH originating from donor cells. We compared the prevalence of CH between recipients and age-matched healthy individuals. A total of 21 recipients and 5183 age-matched controls were analyzed (52.9 ± 11.3 years vs. 54.2 ± 8.1 years, *p* = 0.614). CH was observed in 6 (28.6%) of the recipients and 605 (11.7%) of the age-matched controls with a VAF threshold of 1.5%. With a VAF threshold of 2.0%, CH was observed in 5 (23.8%) of the recipients and 463 (8.9%) of the age-matched controls. These results indicate that the prevalence of CH originating from donor cells was significantly higher in post-allo-HSCT recipients than in the control group (*p* = 0.030 and 0.035 for VAF thresholds of 1.5% and 2.0%, respectively).

Mutations in recipients involved genes such as *DNMT3A*, *BCOR*, *TET2*, *NOTCH2*, and *TP53*. After adjusting for age and sex, *DNMT3A* showed the most significant association with post-allo-HSCT donor-originated CH, with adjusted odds ratios of 3.652 and 4.923 for VAF thresholds of 1.5% and 2.0%, respectively. The 95% confidence intervals (CI) were [1.192–11.189] (*p* = 0.0234) and [1.594–15.199] (*p* = 0.0056), respectively (Table S2).

### 2.5. Telomere Length

The median the telomere-to-single copy gene (T/S) ratio of recipients before HSCT was lower than that of the donor [median 0.56 (range 0.31–1.01) vs. 0.915 (range 0.54–1.69), *p* < 0.001 by Mann–Whitney U test]. After HSCT, the median T/S ratio of recipients was 0.83 (range 0.35–1.13), significantly higher than that before HSCT (*p* = 0.027 by Mann–Whitney U test).

The Wilcoxon signed-rank test revealed that the T/S ratio of transplanted cells into recipients was significantly lower than their original cells (*p* = 0.010). Hematopoietic cells sustained in the donor’s body also showed a reduction in the T/S ratio after HSCT (*p* = 0.005), possibly reflecting an aging phenomenon, notably higher than that of transplanted cells (*p* = 0.021) (Figure 2). This indicates accelerated telomere shortening when healthy donor cells are transplanted into the recipient compared to their persistent existence in the donor’s body for the same period.

## 3. Discussion

CH is characterized by recurrent mutations in known oncogenes within hematopoietic cells, occurring without evident hematologic malignancies [10]. Despite the increased prevalence of CH with age, CH mutations tend to remain relatively stable over time in the absence of additional stressors [11]. This stability suggests that extrinsic factors provide opportunities for somatic mutations to evolve, leading to the selection and expansion of clones within specific microenvironments [12]. Alterations in the BM microenvironment can disrupt the normal development and fate of HSCs [6]. Our study observed an increased occurrence of somatic mutations in recipients following allo-HSCT, consistent with findings in animal models [13,14]. These somatic mutations were classified as CH due to their low burden and lack of association with malignant transformation. Notably, instances of CH primarily originated from donor cells, as evidenced by sustained complete donor chimerism without any disease recurrence. A notable increase in CH development compared to age-matched controls suggests potential influences from transplant procedures on hematopoietic cells and/or BM microenvironments. Additionally, the lack of similar changes in hematopoietic cells aging in the donor’s body over the same duration supports the notion that these effects are more likely attributable to these factors than age influences on CH.

The results from telomere length measurements were particularly intriguing. Although the telomere length of the donor cells decreased in the donor’s body over time, the shortening was potentiated when they were transplanted into recipients over the same period. Previous studies have demonstrated telomere shortening after HSCT, with the degree of telomere loss equivalent to a median of 15 years of aging in healthy controls [15,16]. Particularly during engraftment and in the first post-transplant year, telomere shortening is primarily a consequence of the extensive cell proliferation needed to achieve immune reconstitution [17,18]. It is possible that replicative senescence occurs during the extensive cell proliferation in HSC, accelerating the shortening of telomere length in donor cells, as observed in MSC [19]. Additionally, replicative senescence has been associated with the accumulation of genomic instability [20], which may eventually lead to the development of CH, as demonstrated in our study.

In HSCT, CH is a highly prevalent condition in both allo-HSCT recipients and autologous HSCT [21]. The persistence of CH in transplanted donor cells has long been noted, usually without detriment to recipients’ survival [22]. However, a recent study unveiled varying effects on graft alloimmune function and the potential for leukemic transformation, linked to the mutated gene and somatic clonal abundance [23]. CH may also emerge de novo from donor cells after allo-HSCT [24]. In this study, the majority of post-allo-HSCT CH cases were de novo, originating from donor cells, and did not coincide with somatic mutations present before HSCT. This suggests that microenvironmental changes capable of generating an inflammatory environment may facilitate the acquisition and selection of mutations, rather than the persistence of an MDS-causing microenvironment following HSCT. Our findings on mutational types align with previous studies, indicating that *DNMT3A* and *TET2* are the most commonly mutated genes [25,26]. *DNMT3A* emerges as the predominant gene in allo-HSCT-associated CH, implicated not only in aging but also in chronic infection through IFNγ signaling inflammation [27,28]. *TET2*, another frequently mutated gene in allo-HSCT CH, exhibits an association between increased Tet2+/− clonal expansion and elevated levels of the inflammatory cytokine interleukin-1 in the BM during aging [29]. While the *BCOR* gene and its homolog *BCORL1* are implicated in various hematologic malignancies and aplastic anemia, their association with allo-HSCT is rarely reported. However, there is a documented case of a patient with a *BCORL1* mutation, acquired or selected after HSCT, coinciding with the onset of severe peripheral artery disease [30]. Furthermore, CH involving DNA damage response genes, such as *TP53* and *PPM1D*, has been reported in transplant recipients, particularly those undergoing autologous HSCT following prior cytotoxic therapy [31,32]. Our results showed that the mutations identified before HSCT were not affected by bridging chemotherapy, although it was unclear if those mutations resulted from therapy. While the significance of these CH occurrences in allo-HSCT remains uncertain, they suggest the possibility of considering such cases as high-risk CH [24].

We acknowledge our inability to identify the exact stressor inducing the development of post-allo-HSCT CH, as it was not associated with any preexisting factors. The significance of post-allo-HSCT CH was not explicitly suggested in our study. Despite these limitations, our investigation initiates a crucial dialogue regarding the necessity of monitoring CH in post-allo-HSCT. This study sheds light on the importance of recognizing the higher incidence of CH in donor cells transplanted into recipients. This insight stems from a direct comparison with cells remaining in the donor’s body, as well as a comparison with age-matched controls. Furthermore, the accelerated telomere shortening in these cells presents partial evidence of increased genomic instability. This phenomenon is attributed to replicative senescence caused by accelerating proliferation stress of HSCs, as well as the microenvironmental changes resulting from transplantation-associated stress, including preconditioning such as irradiation and cytotoxic regimens, which create an inflammatory environment.

## 4. Materials and Methods

### 4.1. Study Population

A total of 21 recipients diagnosed with MDS who underwent allo-HSCT between September 2009 and February 2015 were enrolled, meeting inclusion criteria of full donor chimerism and a disease-free status. To compare the occurrence of CH before and after HSCT, we utilized DNA samples isolated from PB of recipients. DNA samples from corresponding HSCT donors were obtained after obtaining informed consent.

As a control group representing CH in the general population, we used data from participants in the GENIE cohort who underwent routine health checkups at the Seoul National University Hospital Healthcare System Gangnam Center between January 2014 and January 2017. Details of the cohort have been described previously [33]. From the GENIE cohort, we included age-matched participants whose blood samples were sequenced for CH.

### 4.2. Identification of Mutations Associated with CH

#### 4.2.1. Sequencing

Targeted sequencing was performed using a Twist Library Preparation EF kit with a custom panel and Illumina NovaSeq 6000 (Illumina, Inc., San Diego, CA, USA) with 2 × 150 bp paired-end reads. The custom panel comprised the total exon of 26 genes, including *ASXL1*, *ATM*, *BCOR*, *CBL*, *CHEK2*, *CREBBP*, *DNMT3A*, *EP300*, *GNAS*, *IDH2*, *JAK2*, *KMT2C*, *KMT2D*, *KRAS*, *NOTCH1*, *NOTCH2*, *PPM1D*, *SETD2*, *SF3B1*, *SH2B3*, *SRSF2*, *STAG2*, *STAT3*, *TET2*, *TP53*, and *U2AF1*.

#### 4.2.2. Data Preprocessing, Quality Control Analysis, and Control Cohort

Targeted sequencing reads from PB samples were demultiplexed using Illumina’s bcl2fastq (v2.17.1.14) to generate FASTQ files. We employed SeqPrep for adapter trimming (default settings) and Sickle (v1.33) for low-BQV base trimming (minimum average BQV = 20). Subsequently, trimmed FASTQ files underwent the GATK best practice pipeline, involving alignment to the hg19 reference with BWA-MEM (v0.7.10). For all samples, duplicate marking and sorting were conducted using PICARD (v1.94) MarkDuplicates, followed by indel realignment and base quality score recalibration using GATK Light (v2.3.9). Duplicate marking was repeated, resulting in a final coordinate-sorted BAM per sample, ready for analysis. Duplication metrics and BAM quality metrics were computed using PICARD (v1.94; MarkDuplicates, CalculateHsMetrics, CollectGcBiasMetrics). To filter out artifactual variants, we utilized a CHIP negative healthy cohort (*n* = 209, aged 20–30 years). Analysis-ready BAM files for the analyzed cohort and CHIP-negative cohort were qualified with a depth of coverage (average DOC > 1000×), ensuring a 2% VAF limit of detection.

#### 4.2.3. Somatic Mutation Calling and Filtering

The analysis-ready BAM files underwent a somatic variant calling pipeline, including VarDict (1.8.2), Mutect2 (4.1.4.1), and SNVer (0.4.1), for SNV, insertion, and deletion calling. To ensure comprehensive somatic variant calling, we combined results from all SNV callers. Specifically, positive SNVs/indels were required to have total reads >= 20, Alt reads >= 10 (positive and negative >= 5, respectively), and a VAF between 1% and 30%. Variants with VAF > 30% were considered if they met COSMIC hematological criteria and had a median batch VAF < 45%. Additionally, we filtered common germline variants with a minor allele frequency (MAF) > 0.1% in gnomAD. Subsequently, a subset of artifactual calls with MAF > 2% in somatic variants was filtered out using data from the 209 CHIP-negative cohort, which did not match COSMIC hematological criteria. Final variants underwent curation by IGV review to eliminate potential artifacts driven by PCR, highly homologous regions, and repeat regions.

### 4.3. Telomere Length Analysis

Telomere length was measured using real-time quantitative RT-PCR, as described previously [19]. Telomere length is determined by the T/S ratio, which has been shown to be proportional to the average telomere length in a cell.

### 4.4. Statistical Analysis

The data are presented as the median and range. Group comparisons were conducted using the Mann–Whitney U test, and paired comparisons were performed using the Wilcoxon signed-rank test. Significance was set at * *p* < 0.05 and ** *p* < 0.01. Statistical analyses were conducted using IBM^®^ SPSS^®^ Windows, Version 24.0 (IBM Corp., Armonk, NY, USA).

The prevalence levels of CH in MDS patients and the GENIE cohort were compared using the χ2 test. Multivariate logistic regression, adjusted for age and sex, was used to identify independent risk factors for each CH at VAF thresholds of 1.5% and 2.0% in both the MDS patients and the GENIE cohort. Statistical analyses were performed using R statistical software, version 4.0.2 (http://www.r-project.org).

## 5. Conclusions

In conclusion, our findings emphasize the need for continued research and careful monitoring of CH post-allo-HSCT. This enhances our understanding of the intricate interactions among genetic, environmental, and therapeutic elements influencing CH development and progression in transplant recipients, alongside the clinical and prognostic implications of post-allo-HSCT CH.

## Figures and Tables

**Table 1:** Baseline characteristics of recipients and transplantation details.

Cases	Sex	Age (Years)		Bridging Chemotherapy	Worst Subtypes	Donor	CI	aGVHD	cGVHD	GCV	Sampling Time	Relapse	Survival
		R	D								Post-Transplant Years		
R1	F	26	20	none	MDS-EB−1	MSD	RIC	none	none	none	5.2	none	alive (11.9)
R2	M	43	39	DEC	MDS-EB−1	MSD	MAC	1	severe	none	6.7	none	alive (13.4)
R3	M	30	67	DEC	MDS/AML	HID	MAC	2	moderate	none	6.0	none	alive (12.6)
R4	M	64	64	none	MDS-EB−1	MSD	RIC	2	mild	none	6.1	none	alive (12.1)
R5	M	41	46	DEC	MDS-EB−2	HID	MAC	2	severe	none	5.8	none	dead (8.3)
R6	F	38	36	AZA	MDS-EB−1	MSD	MAC	2	none	none	2.5	relapse (10.2)	alive (10.3)
R7	F	37	39	DEC	MDS-MLD	MSD	RIC	0	mild	none	5.4	none	alive (12.1)
R8	F	42	38	DEC	MDS-EB−1	MSD	MAC	none	severe	yes	7.0	none	alive (13.3)
R9	M	62	24	AZA + DEC	MDS/AML	HID	MAC	2	moderate	none	7.2	none	alive (13.6)
R10	F	55	46	AZA	MDS-EB−2	MSD	RIC	none	moderate	none	8.3	none	alive (14.5)
R11	M	47	18	AZA	MDS-MLD	HID	MAC	none	moderate	none	7.2	none	alive (13.5)
R12	M	54	49	none	MDS-EB−1	MSD	RIC	2	none	none	4.0	none	alive (11.1)
R13	M	44	46	none	MDS-EB−1	MSD	RIC	none	mild	none	5.1	none	alive (11.8)
R14	M	41	36	none	MDS-MLD	MSD	MAC	none	none	none	8.4	none	alive (14.4)
R15	M	33	36	none	MDS-EB−1	MSD	MAC	none	none	none	8.3	none	alive (14.4)
R16	M	40	38	none	MDS-EB−2	HID	MAC	none	none	none	6.0	none	alive (12.3)
R17	M	58	26	AZA	MDS-EB−2	MUD	MAC	none	none	none	3.0	none	alive (9.0)
R18	M	59	55	AZA	MDS-U	MSD	RIC	2	moderate	none	5.2	none	alive (11.8)
R19	M	56	20	none	MDS-EB−1	HID	RIC	none	none	none	5.6	none	alive (11.7)
R20	M	64	59	AZA	MDS-EB−2	MSD	MAC	3	mild	none	6.1	none	alive (12.1)
R21	F	50	43	DEC	MDS-MLD	MSD	RIC	4	severe	none	5.6	none	alive (12.3)

Abbreviations: R, recipient; D, donor; DEC, decitabine; AZA, azacytidine; MDS, myelodysplastic syndrome; AML, acute myeloid leukemia; EB, excess blasts; MLD, multilineage dysplasia; U, unclassifiable, MSD, matched sibling donor; HID, haploidentical donor; MUD, matched unrelated donor; CI, conditioning intensity; RIC, reduced intensity conditioning; MAC, myeloablative conditioning; aGVHD, acute graft-versus-host disease; cGVHD, chronic graft-versus-host disease; GCV, ganciclovir treatment.

## Data Availability

The datasets generated during and/or analyzed during the current study are available from the corresponding author on reasonable request.

## Supplementary Materials

The supporting information can be downloaded at https://www.mdpi.com/article/10.3390/ijms251910258/s1.
