# Supplementary material for: Influence of the Bone Marrow Microenvironment on Hematopoietic Stem Cell Behavior Post-Allogeneic Transplantation: Development of Clonal Hematopoiesis and Telomere Dynamics
Source: Int J Mol Sci. 2024 Sep 24;25(19):10258. doi: 10.3390/ijms251910258 (PMC11477089; doi:10.3390/ijms251910258)
Supplement: Supplementary file 1 [file ijms-25-10258-s001.zip › Table S1.pdf]

Table S1. Genetic mutations

Sample	Gene	Chromosome	Position	Variant classification	Reference allele	Alternate allele	Protein change	CDS	VAF
R_PRE	*ASXL1*	chr20	31022286	STOP_GAINED	T	TA	p.590Tyr_591GlninsTer???	c.1772_1773insA	40.17
R_PRE	*ASXL1*	chr20	31022402	FRAME_SHIFT	TCACCACTGCCATAGAGAGGCGGC	T	p.629His_637Thrfs	c.1888_1896delCACCACTGCCATAGAGAGGCGGC	1.28
R_PRE	*ASXL1*	chr20	31022484	STOP_GAINED	G	T	p.Glu657*	c.1969G>T	10.9
R_PRE	*ASXL1*	chr20	31022539	FRAME_SHIFT	C	CCTCA	p.674Pro_675Glufs	c.2025_2026insCTCA	4.29
R_PRE	*ASXL1*	chr20	31022837	FRAME_SHIFT	AT	A	p.774Leu_775Valfs	c.2323_2324delT	24.32
R_PRE	*ASXL1*	chr20	31023011	FRAME_SHIFT	CA	C	p.832Ser_833Hisfs	c.2497_2498delA	4.24
R_PRE	*ASXL1*	chr20	31023271	FRAME_SHIFT	T	TA	p.918Ile_919Profs	c.2757_2758insA	27.98
R_PRE	*ASXL1*	chr20	31023408	STOP_GAINED	C	T	p.Arg965*	c.2893C>T	4.67
R_PRE	*ASXL1*	chr20	31024758	STOP_GAINED	C	T	p.Arg1415*	c.4243C>T	5.2
R_PRE	*BCOR*	chrX	39922030	FRAME_SHIFT	CCT	C	p.1379Thr_1381Glufs	c.4140_4142delGA	7.69
R_POST	*BCOR*	chrX	39933112	FRAME_SHIFT	AT	A	p.495Ile_496Tyrfs	c.1486_1487delA	1.48
R_POST	*BCOR*	chrX	39933138	FRAME_SHIFT	T	TA	p.486Leu_487Serfs	c.1460_1461insT	2
R_PRE	*BCOR*	chrX	39933311	STOP_GAINED	G	A	p.Gln430*	c.1288C>T	2.38
R_PRE	*CHEK2*	chr22	29085161	NON_SYNONYMOUS_CODING	C	T	p.Glu545Lys	c.1633G>A	1.2
R_PRE	*CHEK2*	chr22	29085164	STOP_GAINED	C	A	p.Glu544*	c.1630G>T	1.3
R_POST	*DNMT3A*	chr2	25457242	NON_SYNONYMOUS_CODING	C	T	p.Arg882His	c.2645G>A	5.88
R_POST	*DNMT3A*	chr2	25457242	NON_SYNONYMOUS_CODING	C	T	p.Arg882His	c.2645G>A	1.62
R_POST	*DNMT3A*	chr2	25458688	STOP_GAINED	T	A	p.Lys829*	c.2485A>T	4.78
R_POST	*DNMT3A*	chr2	25466778	NON_SYNONYMOUS_CODING	C	G	p.Gly642Ala	c.1925G>C	4.22
R_POST	*DNMT3A*	chr2	25467023	SPLICE_SITE_DONOR	C	G	-	-	9.07
R_POST	*DNMT3A*	chr2	25467105	FRAME_SHIFT	AC	A	p.589Gly_590Thrfs	c.1769_1770delG	2.3
R_PRE	*DNMT3A*	chr2	25467485	NON_SYNONYMOUS_CODING	C	T	p.Asp531Asn	c.1591G>A	25.23
R_PRE	*DNMT3A*	chr2	25467495	NON_SYNONYMOUS_CODING	C	A	p.Gln527His	c.1581G>T	1.32
R_PRE	*GNAS*	chr20	57484421	NON_SYNONYMOUS_CODING	G	A	p.Arg844His	c.2531G>A	3.5
R_PRE	*IDH2*	chr15	90631934	NON_SYNONYMOUS_CODING	C	T	p.Arg140Gln	c.419G>A	4.78
R_PRE	*KMT2D*	chr12	49441838	FRAME_SHIFT	T	TA	p.1381Val_1382Cysfs	c.4145_4146insT	1.14
R_POST	*NOTCH2*	chr1	120480600	NON_SYNONYMOUS_CODING	T	C	p.Lys1073Glu	c.3217A>G	1.77
R_POST	*PPM1D*	chr17	58740749	STOP_GAINED	C	T	p.Arg552*	c.1654C>T	1.52
D_PRE	*PPM1D*	chr17	58740749	STOP_GAINED	C	T	p.Arg552*	c.1654C>T	1.18
R_PRE	*SF3B1*	chr2	198266713	NON_SYNONYMOUS_CODING	C	T	p.Gly740Glu	c.2219G>A	1.57
R_PRE	*SF3B1*	chr2	198266831	NON_SYNONYMOUS_CODING	C	A	p.Val701Phe	c.2101G>T	21.13
R_PRE	*SF3B1*	chr2	198266834	NON_SYNONYMOUS_CODING	T	C	p.Lys700Glu	c.2098A>G	14.6
R_PRE	*SRSF2*	chr17	74732935	CODON_CHANGE_PLUS_CODON_DELETION	CGGCGGCTGTGGTGTGAGTCCGGGG	C	p.94Pro_102Argdel	c.284_292delCCGCCGACACCACACTCAGGCCCC	29.97
R_PRE	*STAG2*	chrX	123224615	SPLICE_SITE_DONOR	G	GT	-	-	4.29
R_POST	*TET2*	chr4	106155778	FRAME_SHIFT	G	GA	p.226Glu_227Lysfs	c.680_681insA	1
R_POST	*TET2*	chr4	106157589	FRAME_SHIFT	ACAGGTTT	A	p.830Gln_833Cysfs	c.2491_2494delCAGGTTT	2.25
R_PRE	*TET2*	chr4	106158018	STOP_GAINED	C	A	p.Cys973*	c.2919C>A	3.18
R_PRE	*TET2*	chr4	106190851	NON_SYNONYMOUS_CODING	T	A	p.Phe1377Ile	c.4129T>A	1.2
R_PRE	*TET2*	chr4	106193802	STOP_GAINED	A	T	p.Lys1422*	c.4264A>T	7
R_POST	*TP53*	chr17	7577114	NON_SYNONYMOUS_CODING	C	T	p.Cys275Tyr	c.824G>A	1.8
R_PRE	*TP53*	chr17	7578190	NON_SYNONYMOUS_CODING	T	C	p.Tyr220Cys	c.659A>G	1.03
R_PRE	*U2AF1*	chr21	44524456	NON_SYNONYMOUS_CODING	G	A	p.Ser34Phe	c.101C>T	16.5
