# Supplementary material for: Influence of the Bone Marrow Microenvironment on Hematopoietic Stem Cell Behavior Post-Allogeneic Transplantation: Development of Clonal Hematopoiesis and Telomere Dynamics
Source: Int J Mol Sci. 2024 Sep 24;25(19):10258. doi: 10.3390/ijms251910258 (PMC11477089; doi:10.3390/ijms251910258)
Supplement: Supplementary file 1 [file ijms-25-10258-s001.zip › Table S2.pdf]

Table S2. Adjusted odd ratio for prediction of clonal hematopoiesis

	**aOR**	**95% Confidence Interval**	***P***
DNMT3A 1.5	3.652	1.192 - 11.189	0.0234
DT 1.5	2.905	0.944 - 8.937	0.0629
DTA 1.5	2.607	0.846 - 8.030	0.0950
DTP 1.5	2.858	0.927 - 8.809	0.0675
DTAP 1.5	2.568	0.832 - 7.925	0.1009
CH 1.5	3.492	1.311 - 9.304	0.0124
DMNT3A 2.0	4.923	1.594 - 15.199	0.0056
DT 2.0	3.934	1.270 - 12.187	0.0176
DTA 2.0	3.475	1.121 - 10.768	0.0309
DTP 2.0	3.893	1.255 - 12.080	0.0186
DTAP 2.0	3.442	1.109 - 10.683	0.0324
CH 2.0	3.707	1.308 - 10.504	0.0137

Abbreviations: aOR, adjusted odd ratio; 1.5, 1.5% threshold for variant allele frequency; DT, *DNMT3A* & *TET2*; DTA, DT & *ASXL1*; DTP, DT & *PPM1D*; DTAP, DTA & *PPM1D*; CH, clonal hematopoiesis; 2.0, 2.0% threshold for variant allele frequency
